# Humanized DRAGA mice immunized with *Plasmodium falciparum* sporozoites and chloroquine elicit protective pre-erythrocytic immunity

**DOI:** 10.1186/s12936-018-2264-y

**Published:** 2018-03-14

**Authors:** Sai Majji, Wathsala Wijayalath, Soumya Shashikumar, Teodor D. Brumeanu, Sofia Casares

**Affiliations:** 1 US Military Malaria Vaccine Program, Naval Medical Research Center/Walter Reed Army Institute of Research, Silver Spring, MD USA; 2 Department of Medicine, Uniformed Services University of Health Sciences, Bethesda, MD USA

**Keywords:** Humanized DRAGA mice, *Plasmodium falciparum*, Malaria, Human T cells, Antibodies, Chloroquine, Immunization

## Abstract

**Background:**

Human-immune-system humanized mouse models can bridge the gap between humans and conventional mice for testing human vaccines. The HLA-expressing humanized DRAGA (HLA-A2.HLA-DR4.Rag1KO.IL2RγcKO.NOD) mice reconstitute a functional human-immune-system and sustain the complete life cycle of *Plasmodium falciparum*. Herein, the DRAGA mice were investigated for immune responses following immunization with live *P. falciparum* sporozoites under chloroquine chemoprophylaxis (CPS-CQ), an immunization approach that showed in human trials to confer pre-erythrocytic immunity.

**Results:**

The CPS-CQ immunized DRAGA mice (i) elicited human CD4 and CD8 T cell responses to antigens expressed by *P. falciparum* sporozoites (Pfspz) and by the infected-red blood cells (iRBC). The Pfspz-specific human T cell responses were found to be systemic (spleen and liver), whereas the iRBCs-specific human T cell responses were more localized to the liver, (ii) elicited stronger antibody responses to the Pfspz than to the iRBCs, and (iii) they were protected against challenge with infectious Pfspz but not against challenge with iRBCs.

**Conclusions:**

The DRAGA mice represent a new pre-clinical model to investigate the immunogenicity and protective efficacy of *P. falciparum* malaria vaccine candidates.

**Electronic supplementary material:**

The online version of this article (10.1186/s12936-018-2264-y) contains supplementary material, which is available to authorized users.

## Background

Malaria is a deadly infectious disease caused by a protozoan of the *Plasmodium* species. The disease is initiated by the bite of an infected female *Anopheles* mosquito and inoculation of sporozoites in skin, which rapidly migrate through the bloodstream to infect hepatocytes. Mature liver stage parasites are then released to the bloodstream to invade the red blood cells and to initiate the asexual erythrocytic cycles responsible for the clinical manifestations of malaria [1]. Among the five species of *Plasmodium* that infect humans, *Plasmodium falciparum* is the most virulent with 212 million new cases worldwide, and 429,000 deaths reported in the year of 2016 mainly infants, children, and pregnant women living in sub-Saharan Africa [2].

Rodents, New World monkeys, and chimpanzees have been used for decades as pre-clinical models of malaria. While rodents have been critically important to study the biology of malaria parasites, it is now clear that rodent malaria parasites do not represent the complexity of *P. falciparum*. First, the duration of liver stage infection is 2–3 days for rodent malaria parasites, but 7 days for *P. falciparum* [3]. Secondly, rodent malaria parasites lack orthologues for many proteins expressed by *P. falciparum* such as the liver stage antigen 1 (LSA1) [4] and *var, rif, stevor*, and *Pfmc*-*2TM*, which are responsible for immune evasion [1]. Thirdly, proteins shared by *P. falciparum* and rodent malaria parasites differ in their biological function [5]. New World monkeys can be experimentally infected with monkey-adapted *P. falciparum* blood stage parasites, and splenectomy is required for long-term blood infections [6]. Except for one Aotus subspecies (*Aotus lemurinus griseimembra*), New World monkeys do not sustain reproducible *P. falciparum* liver stage infection [6]. Great apes can be experimentally infected with *P. falciparum* sporozoites or with blood stage parasites, but the use of great apes for research is under moratorium [7]. Consequently, the lack of convenient animal models for *P. falciparum* has urged the need of testing the protective efficacy of human malaria vaccine candidates directly in human trials.

HLA class II-expressing DRAG (HLA-DR4.RagKO.IL2RγcKO.NOD) mice infused with HLA-matched human haematopoietic stem cells (HSC) efficiently repopulate the mouse thymus with human T cell precursors, and reconstitute peripheral lymphoid organs with functional human T cells and with human B cells that undergo immunoglobulin class switching and secrete human IgG [8–12]. The DRAG mice, by virtue of reconstituting human cell compartments, sustain infection with human pathogens, such as *P. falciparum*, HIV-1, Zika, and influenza A viruses [9–13]. Since DRAG mice do not express transgenically human HLA class-I molecules, and expression of HLA class-I molecules is required for reconstitution of HLA class-I restricted human CD8 T cells, HLA class I/II-expressing DRAGA (HLA-A2.HLA-DR4.RagKO.IL2RγcKO.NOD) mice were generated and upon vaccination they showed to elicit human CD8 T cells that are HLA class I-restricted and cytotoxic [14].

Immunization of humans with *P. falciparum* sporozoites under chloroquine prophylaxis (CPS-CQ) elicits pre-erythrocytic immunity [15–19], since the immunized volunteers were protected against challenge with *P. falciparum* sporozoites (Pfspz), but not against challenge with *P. falciparum* infected-red blood cells (iRBCs) [19]. To determine whether the human immune system of DRAGA mice is competent enough to elicit protective immune responses against malaria parasites, DRAGA mice were immunized with *P. falciparum* CPS-CQ. The immunized DRAGA mice elicited human T cell and antibody responses to the Pfspz and to the iRBCs, and they were protected against challenge with Pfspz, but not against challenge with iRBCs. The results indicate the potential of DRAGA mice to investigate the immunogenicity and protective efficacy of *P. falciparum* malaria vaccine candidates.

## Methods

### Mice

DRAGA mice express HLA-A2.1 and HLA-DR0401 molecules on a Rag1KO.IL2RγcKO.NOD (NRG) background and they have been previously described [14]. HLA-A2.1.HLA-DR0401 positive umbilical cord bloods were obtained from the NY Blood Center, Long Island City. Four to six-week old DRAGA mice were irradiated (350 rads) and injected intravenously with CD3 T cell-depleted cord blood cells (EasySep Human CD3 Positive Selection Kit, Stem Cell Technologies, #18051) containing approximately 10^5^ human HSC (CD34^+^) as measured by FACS using human CD34 antibodies (clone#563, BDbiosciences). The procedures for assessing human T and B cell and erythrocyte reconstitution in peripheral blood by FACS have been previously described [8–10, 14]. Human albumin plasma levels were measured by ELISA (Human albumin ELISA kit, Bethyl Labs). DRAGA mice were used at 4 months post-infusion of human HSC. As previously reported [14] most (> 90%) DRAGA mice reconstitute human cells. The human reconstitution status in blood of DRAGA mice used in this study is shown in Additional file 1: Table S1.

### Parasites

*Plasmodium falciparum* (NF54)-infected *Anopheles stephensi* mosquitoes were obtained from the Department of Entomology at WRAIR/NMRC. The Pfspz were isolated by dissecting the salivary glands as described [10]. Cultures of *P. falciparum* infected red blood cells (iRBCs) (NF54) containing a mixture of asexual stage parasites (rings, trophozoites and schizonts) were obtained from the Malaria Culture Core at WRAIR/NMRC.

### CPS-CQ immunization

DRAGA mice were injected intravenously with Pfspz (NF54, 10^5^/mouse) three times at 2 weeks apart. During each immunization mice were injected with chloroquine diphosphate (25 mg/kg weight, intraperitoneally) at days 0, 1 and 7 of the sporozoite injection. Three weeks after the last immunization mice were either euthanized to assess human T cell responses in spleen and liver, or they were challenged with *P. falciparum* parasites.

### T cell assays

Splenic cells were isolated as described [8–10]. For isolation of liver mononuclear cells, the livers were disrupted as described [10], spun at 60 g for 1 min to remove hepatocytes, and the supernatants were further spun at 890 g for 10 min. Cell pellets were suspended in PBS and subjected to 36% Percoll density gradient (Percoll, Sigma-Aldrich, St. Louis, MO) centrifugation at 890*g* for 30 min for isolation of mononuclear cells. Splenic cells and liver mononuclear cells were cultured in 96 flat-well plates (5 × 10^5^ per well/0.2 ml) and stimulated either with irradiated Pfspz (10,000 rads, 25,000 per well), with iRBCs protein extracts (200 µg/ml**),** with PfAMA1_406–414_ (TQKCEIFNV) (10 µg/ml) synthetic peptide (Mimotopes) [20] for 4 days, or with PMA (1 µg/ml) plus ionomycin (50 nM) (Sigma) for 2 days. Control cultures were non-stimulated, or they were stimulated with un-infected RBC (uRBC) protein extracts for 4 days. Human cytokines secreted in cell culture supernatants were measured by Luminex (#LHC0001M, InVitrogen), which is human validated and does not cross-react with mouse cytokines [8, 14].

### *Plasmodium falciparum* iRBCs protein extracts

Cultures of *P. falciparum* iRBCs (NF54), or equal numbers of uninfected RBCs, were pelleted and suspended in ACK for 5 min on ice to lyse the erythrocytes and washed twice in 1×PBS (4000 rpm, 10 min). The pellet was freeze-thawed three times using liquid nitrogen and boiling water followed by sonication at 50% amplitude for 20 s with 10 s break for a total cycle of 3 min. Protein concentration was measured by Biuret.

### FACS

Splenic and liver mononuclear cells were incubated for 10 min with Fc Block (BDbiosciences) and surface stained with human CD45 (clone #2D11), CD3 (clone #HIT3a), CD4 (clone #SK3), CD8 (clone #RPA-T8) and CD19 (clone #H1B19) antibodies (BDbiosciences). For intracellular staining, cells were surface stained as above, fixed/permeabilized using BD Cytofix/Cytoperm (BDbiosciences) followed by staining with human FOXP3 (clone #236A/E7, eBioscience), TNFα (clone #Mab11) or IFNγ (clone#B27) antibodies (BDbiosciences) as described [8, 10, 14]. As we previously showed the human antibodies do not cross-react with mouse cells [8, 10, 14]. Cell populations were quantified on the mononuclear FSC/SSC gate (Additional file 2: Figure S1).

### Pfspz and iRBCs antibody titers by IFA

NF54 Pfspz, or cultures of iRBCs at 6% parasitaemia were washed twice in PBS/1% BSA. Teflon printed 12-well slides (Electron Microscopy Sciences, Hatfield, PA) were coated with Pfspz (5000/well) or with iRBC (10,000 RBCs/well at 6% parasitaemia) as described [10]. Slides were air dried and stored at − 80 °C until use. Upon thawing, slides were blocked with PBS/1% BSA for 30 min at 37 °C. Twenty microliters of plasma starting at 1:20 and sequential two-fold serial dilutions were added to the wells and incubated for 1 h at 37 °C. Slides were washed three times with PBS, incubated with FITC-labeled goat F(ab′)_2_ anti-human IgG (γ chain) or anti-human IgM (µ chain) (Southern Biotech) to measure specific human IgG or IgM antibody titers, or with anti-human IgG (H + L) (BDbiosciences) to measure total specific human antibody titers, for 30 min at 37 °C, washed, and mounted with Vectashield-DAPI (Vector Laboratories).

### Parasite challenge

Mice were challenged intravenously with infectious Pfspz (NF54, 10^5^/mouse) or with iRBCs (NF54, 7 × 10^5^/mouse).

### Parasitaemia

Mice challenged with Pfspz were followed for blood stage parasitaemia by PCR as previously described [10]. Briefly, 100 µl of blood was used to extract DNA (DNeasy blood and tissue kits Qiagen, Valencia, CA), and suspended in 100 µl elution buffer. A pair of *Plasmodium* genus-specific primers was used to amplify all units of rRNA distributed in chromosomes 1, 5, 7, 11 and 13; Forward 5-GCTCTTTCTTGATTTCTTGGATG-3 and Reverse 5-AGCAGGTTAAGATCTCGTTCG-3 [10, 21]. PCR was carried out in 20 μl reaction volume containing 5 µl of DNA, 0.025 unit of Taq polymerase (Life technologies), 0.5 μM each primer, 0.2 mM deoxynucleotide triphosphates (dNTPs) (Life technologies) and 0.2 mM MgCl2. PCR settings were: 1 cycle for 95 °C for 10 min followed by 43 cycles of 95 °C for 30 s, 60 °C for 30 s and 72 °C for 1 min. For quality control DNA, samples were PCR using primers specific for HLA-DR4 (housekeeping gene) as described [8]. PCR products were analysed on agarose gels (3%) with ethidium bromide. Quantification of blood stage parasitaemia in mice challenged with iRBCs was carried by qPCR (SYBRgreen, master mix, Applied Biosystems) in 20 μl reaction volume containing 5 µl of DNA and 0.5 μM *Plasmodium* genus-specific primers. The qPCR settings were: 1 cycle of 95 °C for 15 min followed by 45 cycles of 95 °C for 30 s, 60 °C for 30 s. CT values for each sample were plotted against a standard curve that was prepared using 10 fold dilutions of DNA extracted from NF54 synchronized *P. falciparum* ring-stage parasite infected-red blood cells. Specific PCR products were further confirmed by running the qPCR samples in agarose gels (3%) stained with ethidium bromide.

### Statistical analysis

Data were analysed using 2-tailed Student *t* test or Z test at significant level of 0.05. Correlations were assessed by Spearman’s rank correlation coefficient. Non-parametric statistical analyses (mean fold reduction) were also used to analyse data that did not reach statistical power.

## Results

### CPS-CQ immunization alters the numbers of human T and B cells in DRAGA mice

**Fig. 1:**
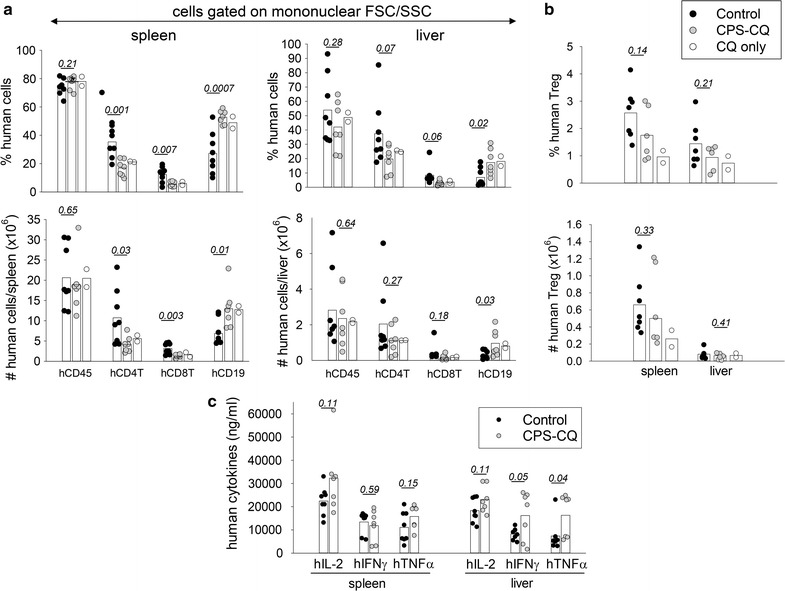
CPS-CQ immunization alters the numbers of human T and B cells in DRAGA mice. DRAGA mice (n = 7) were immunized three times with CPS-CQ at 2 weeks interval, and 3 weeks after the last immunization the splenic cells and liver mononuclear cells were stained with human CD45, CD3, CD4, CD8, CD19, and FOXP3 antibodies and analysed by FACS on the mononuclear FSC/SSC gated cell population. Controls were untreated DRAGA mice (n = 8) or DRAGA mice treated with CQ alone (n = 2). **a** Frequency (upper panels) and total numbers (lower panels) of human CD45, CD4 and CD8 T cells, and B (CD19^+^) cells in spleens (left panels) and liver (right panels). **b** Frequency (upper panel) and total numbers (lower panel) of human CD4^+^FOXP3^+^ regulatory T cells (Treg) in the spleen and liver. Student *t* test p values for comparison of CPS-CQ (n = 7) and control mice (n = 8) in panels A&B are shown over the histograms. The mean average human cell values of two mice treated with CQ alone were similar to mice treated with CPS-CQ and reduced as compared to control mice. **c** Cytokine secretion by splenic and liver mononuclear cells from CPS-CQ immunized and control mice stimulated with PMA/ionomycin. Student *t* test p values are shown over the histograms

*Plasmodium falciparum* CPS-CQ safety trials reported a decrease in the blood leukocyte counts in some of the volunteers after immunization [18]. Thus the effect of CPS-CQ immunization on the numbers of human T and B cells in DRAGA mice was investigated. For this, DRAGA mice (n = 7, Additional file 1: Table S1) were immunized with *P. falciparum* CPS-CQ (10^5^/mouse, three times at 2 weeks interval) and 3 weeks later the numbers of human haematopoietic cells (CD45^+^), human T cell subsets (CD3^+^CD4^+^ and CD3^+^CD8^+^) and human B cells (CD19^+^) in the spleen and liver were measured by FACS. Controls were naïve (untreated) DRAGA mice (n = 8, Additional file 1: Table S1), and DRAGA mice treated with CQ alone (n = 2, Additional file 1: Table S1). As illustrated in Fig. 1a (left panels), the spleens of immunized DRAGA mice contained similar numbers of human haematopoietic cells (CD45^+^) as the control (untreated) DRAGA mice. However, the spleens of immunized DRAGA mice as compared to control (untreated) mice had decreased numbers of human CD4 and CD8 T cells and increased numbers of human B cells (p < 0.05). In the livers of immunized DRAGA mice the mean average number of human CD4 and CD8 T cells was reduced (1.8 and 2.6 fold, respectively) and the mean average for human B cells was increased (2.5 fold) as compared to livers of control DRAGA mice, though the results did not reach statistical significance (Fig. 1a, right panels).

Since regulatory CD4^+^FOXP3^+^ T cells (Tregs) are an important T cell compartment that modulates T cell as well as antibody responses [22], and human Tregs develop in DRAGA mice [14], the effect of CPS-CQ immunization on the numbers of human Tregs was investigated. As illustrated in Fig. 1b, the mean average number of human Tregs in spleen and liver of immunized DRAGA mice was reduced (1.5 fold) as compared to control mice though the results did not reach statistical significance. In aggregate the results indicated that CPS-CQ immunization alters the numbers of human T and B cells. Remarkably, two DRAGA mice treated with CQ alone had human T and B cell mean average numbers that were similar to those in CPS-CQ immunized mice and reduced as compared to the control mice (Fig. 1a, b), suggesting that the lymphocytes changes are a consequence of the CQ treatment rather than to the sporozoite injection.

### CPS-CQ immunization does not affect the cytokine secretory function of human T cells

A critical function of human T cells is cytokine secretion. Cytokines are small molecules that play an important role in regulating humoral and cellular responses [23]. Since CPS-CQ immunization decreased the numbers of human T cells, their functional ability to secrete cytokines was investigated. For this, splenic and liver mononuclear cells from CPS-CQ and control (untreated) DRAGA mice were cultured in vitro with PMA/ionomycin to stimulate the human T cells, and cytokines secreted in the cell culture supernatants were measured by Luminex. As illustrated in Fig. 1c, the splenic and liver T cells from CPS-CQ immunized DRAGA mice were proficient at secreting human IL-2, IFNγ, and TNF. These results showed that while CPS-CQ-immunization decreased the numbers of human T cells, it did not negatively affect their functional ability to secrete cytokines. Indeed, the liver T cells from CPS-CQ-immunized mice secreted higher levels of human IFNγ and TNF than the T cells from control mice, suggesting the presence of activated T cells in the liver of the immunized mice.

### CPS-CQ immunized DRAGA mice elicit human CD4 and CD8 T cell responses to the Pfspz and to the iRBCs

**Fig. 2:**
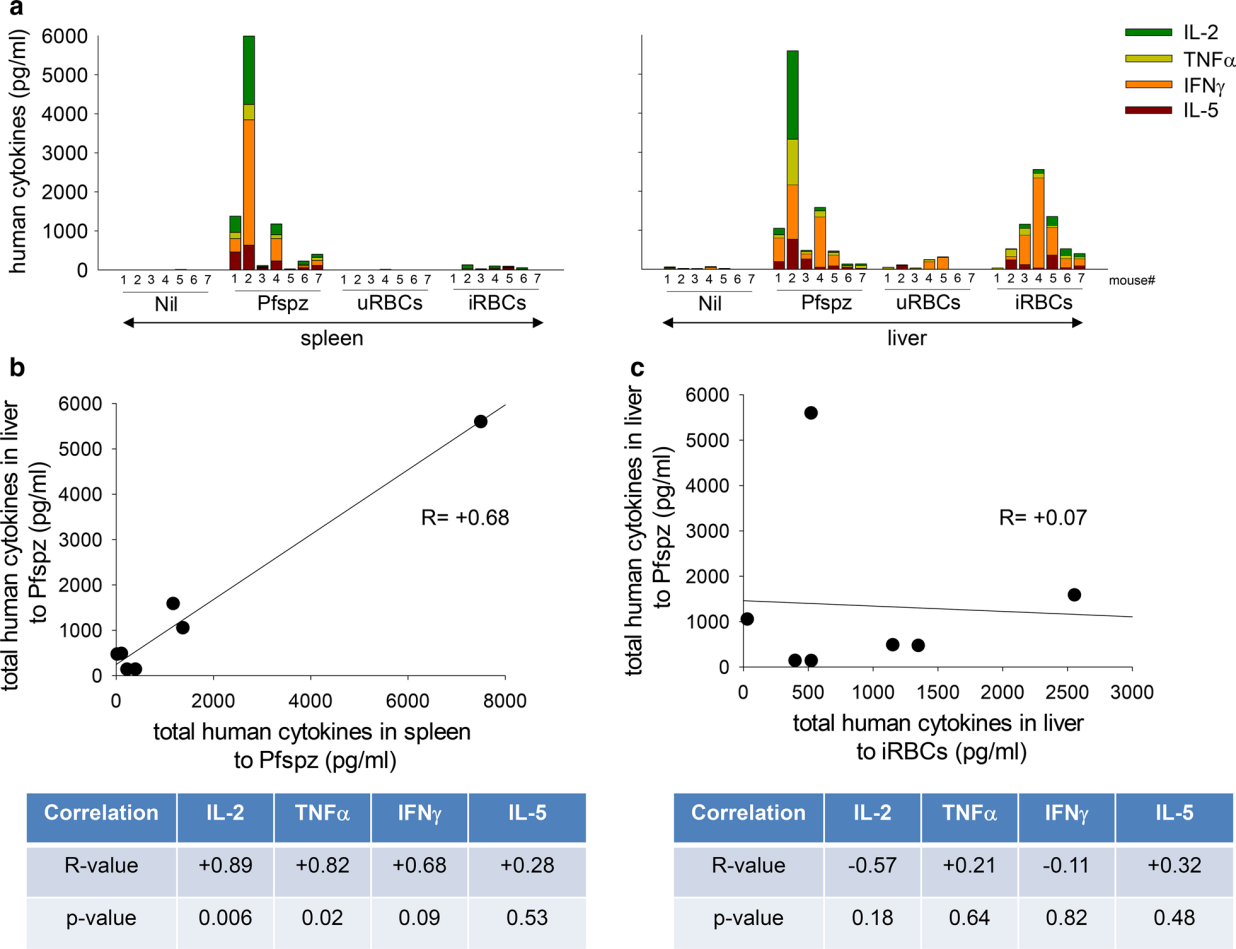
CPS-CQ immunized DRAGA mice elicit human cellular responses to the Pfspz and to the iRBCs. **a** Splenic cells (left) and liver mononuclear cells (right) from immunized DRAGA mice (n = 7) as in Fig. 1, were stimulated for 4 days with Pfspz or with iRBCs (see methods) and human cytokines secreted in cell culture supernatants were measured by Luminex. Control cultures were unstimulated (nil) or stimulated with un-infected RBCS (uRBCs). Bars represent secretion of IL-2, TNF, IFNγ, and IL-5 in individual mice. While splenic and liver mononuclear cells responded to stimulation with Pfspz, only the liver cells responded to stimulation with iRBCs. **b** Spearman correlation for the splenic and liver total human cytokine responses to Pfspz (upper) and for each of the individual human cytokines (IL-2, TNF, IFNγ, IL-5) (lower). **c** Spearman correlation for the total human cytokine responses to the Pfspz and iRBCs within the liver (upper) and for each of the individual human cytokines (IL-2, TNF, IFNγ, IL-5) (lower)

Immunization of humans with *P. falciparum* CPS-CQ allows full liver stage parasite development and transition to the blood stage, as revealed by transient blood stage parasitaemia in most volunteers after the first and second immunizations [15–19]. Thus, the ability of immunized DRAGA mice to elicit cellular immune responses to antigens expressed by the Pfspz and by the iRBCs was investigated. For this, DRAGA mice (n = 7) were immunized with CPS-CQ three times at 2 week intervals. Three weeks after the last immunization, their splenic cells and liver mononuclear cells were stimulated in vitro with Pfspz or with iRBCs, and human cytokines secreted in cell culture supernatants were measured by Luminex. Control cultures were non-stimulated (nil) or stimulated with un-infected red blood cells (uRBCs). As illustrated in Fig. 2a, the splenic and liver cells stimulated with Pfspz secreted human IFNγ, TNF, IL-2, and IL-5 while the non-stimulated cell cultures (nil) failed to secrete cytokines. Furthermore, there was a positive correlation between the total human cytokine response to Pfspz in the spleen and in the liver for each individual mouse (Fig. 2b, upper panel), as well as for each individual human cytokine (IL-2, TNF, IFNγ, IL-5) (Fig. 2b, lower panel). In contrast only the liver mononuclear cells, but not splenic cells, secreted human cytokines upon stimulation with iRBCs. Within the liver, there was no correlation between the human cytokine response to the Pfspz and to the iRBCs (Fig. 2c). In aggregate, these results showed that CPS-CQ immunization induced systemic (spleen and liver) human cellular immune responses to the Pfspz, while human cellular immune responses to the iRBCs were detectable only in the liver.

**Fig. 3:**
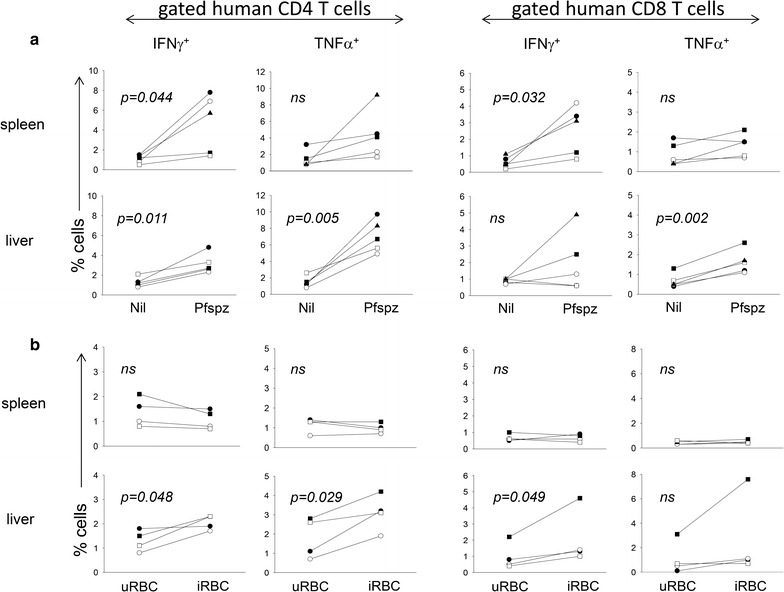
CPS-CQ immunized DRAGA mice elicit human CD4 and CD8 T cells specific for Pfspz and for iRBCs antigens. Splenic cells and liver mononuclear cells from immunized DRAGA mice were stimulated with Pfspz (**a**) or with iRBCs (**b**). Control cultures were unstimulated (nil) or stimulated with un-infected RBC (uRBCs). Cells were stained with human CD3, CD4, CD8, and either IFNγ or TNF Abs and analysed by FACS on the mononuclear FSC/SSC population. Data show frequency of INFγ and TNF secreting cells among gated human CD4 and CD8 T cells. (Blackup-pointing triangle) mice #1&2 (pooled); (Black square) mouse #3, (White square) mice #4&5 (pooled), (Black circle) mouse #6; (White circle) mouse #7. Paired *t* test p values are shown over the histograms, *ns* not significant

The contribution of CD4 and CD8 T cells to the Pfspz and iRBC cytokine response was further investigated. For this, splenic cells and liver mononuclear cells from CPS-CQ immunized DRAGA mice were stimulated for 48 h with Pfspz or with iRBCs and analysed by FACS using human CD3, CD4, CD8, and either IFNγ or TNF antibodies. Control cultures were stimulated with uRBCs or they were non-stimulated (nil). Data depicted in Fig. 3a show that CPS-CQ immunization elicited Pfspz-specific IFNγ^+^CD4 and IFNγ^+^CD8 T cells in the spleen (p < 0.05 paired *t*-test for comparison of all mice) (upper panels), and Pfspz-specific IFNγ^+^CD4, TNF^+^CD4, and TNF^+^CD8 T cells in the liver (p < 0.05) (lower panels). In contrast, T cell responses to iRBCs were detected in the liver, but not in the spleens, and consisted of IFNγ^+^CD4, TNF^+^CD4, and IFNγ^+^CD8 T cells (Fig. 3b). The lack of T responses to the iRBCs in the spleens of immunized DRAGA mice as measured by intracellular FACS was thus in agreement with Luminex data shown in Fig. 2a, which indicated that splenic T cells stimulated with iRBCs failed to secrete cytokines. In summary, these results indicated that CPS-CQ immunized DRAGA mice elicited systemic (spleen and liver) human CD4 and CD8 T cell responses to the Pfspz, while human CD4 and CD8 T cell responses to the iRBCs were mainly localized in the liver.

### AMA1-specific, HLA-A2-restricted human CD8 T cells localize in the liver of CPS-CQ immunized DRAGA mice

**Fig. 4:**
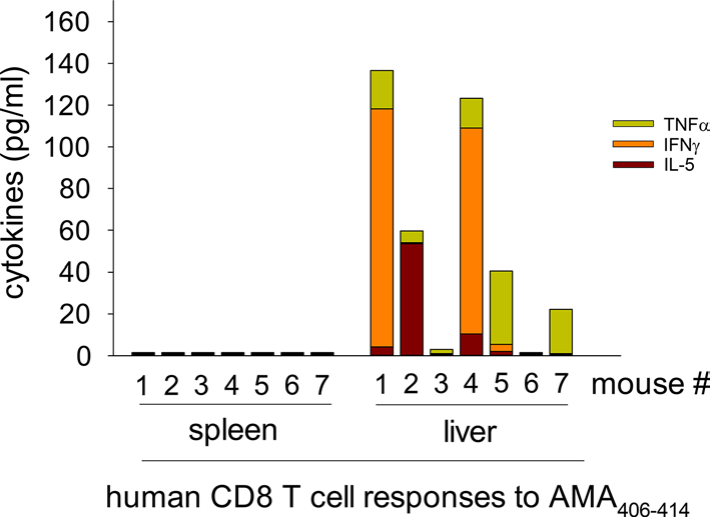
AMA1-specific, HLA-A2-restricted human CD8 T cells localize in the liver of CPS-CQ immunized DRAGA mice. DRAGA mice (n = 7) were immunized with CPS-CQ three times at 2 weeks interval. Three weeks after the last immunization, the cytokine response of splenic and liver human CD8 T cells to stimulation with AMA_406–414_ synthetic peptide was measured by Luminex. Data show values of TNF, IFNγ, and IL-5 in individual mice

Since CD8 T cells play a major role in elimination of malaria-infected hepatocytes [24], the ability of CPS-CQ immunized DRAGA mice to elicit malaria-specific, HLA-A2-restricted human CD8 T cells was investigated. For this, splenic and liver cells from CPS-CQ immunized mice were stimulated in vitro with a synthetic peptide derived from *P. falciparum* apical membrane antigen 1 (AMA1_406–414_) and cytokines secreted in the cell culture supernatants were measured by Luminex. The AMA1_406–414_ peptide is recognized by human CD8 T cells in the context of HLA-A2.1 molecules [20]. As illustrated in Fig. 4, human CD8 T cells in the liver of most immunized mice responded to stimulation with AMA1 synthetic peptide by secreting cytokines (i.e. TNF, INFγ, IL-5) while the splenic human CD8 T cells from the same mice failed to respond to stimulation. These results demonstrated that human AMA1-specific, HLA.A2-restricted CD8 T cells reside mainly in the target organ (liver).

### DRAGA mice immunized with CPS-CQ elicit stronger antibody responses to the Pfspz than to the iRBCs

**Fig. 5:**
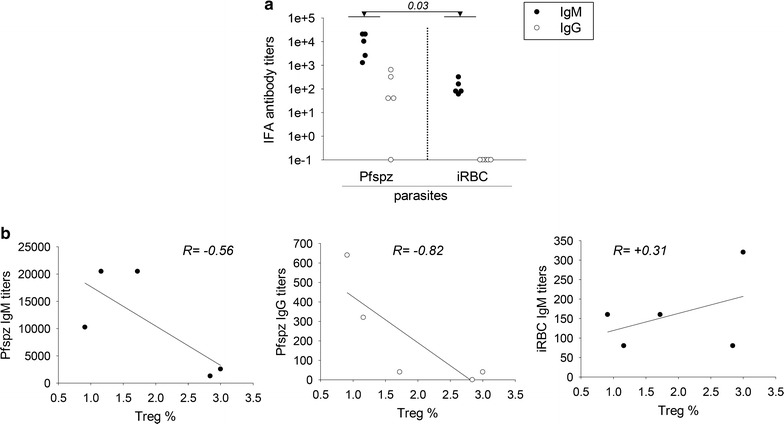
Antibody responses in CPS-CQ-immunized DRAGA mice. **a** DRAGA mice (n = 5, mouse # 3–7) were immunized with CPS-CQ three times at 2 weeks interval. Three weeks after the last immunization serum levels of human IgM and IgG antibodies to Pfspz and iRBCs were measured by IFA. Dots show values in individual mice. Student paired *t* test p value is indicated over the histogram. **b** Human IgM (left) and IgG (middle) antibody titers to the Pfspz negatively correlated with the frequency of human splenic Tregs. In contrast there was no negative correlation between the antibody titers to iRBCs and the Treg frequency (right panel). Spearman’s R values are shown over the histograms

Clinical trials indicated that CPS-CQ-immunized volunteers elicited antibody responses to Pfspz and to a lower extent to the iRBCs [15]. The antibody titers to the Pfspz and iRBCs in CPS-CQ immunized DRAGA mice at day 21 after the third immunization were measured by IFA. As illustrated in Fig. 5a, DRAGA mice elicited higher titers of human IgM antibodies to the Pfspz than to iRBCs (p = 0.03). These mice also elicited human IgG antibodies to the Pfspz while they failed to elicit IgG antibodies to the iRBCs. The results thus indicated that CPS-CQ immunization induced a much stronger antibody response to the Pfspz than to the iRBCs.

### Pfspz antibody titers, but not iRBCs antibody titers, negatively correlate with the frequency of splenic human CD4^+^FOXP3^+^ Tregs

Regulatory CD4^+^FOXP3^+^ T cells (Tregs) regulate humoral responses either by inhibiting the ability of helper CD4 T cells to support B cell antibody secretion [22], or by direct inhibition on the B cells [25]. To determine whether the human Tregs in DRAGA mice can regulate antibody responses to *P. falciparum,* the antibody titers at 3 weeks after the third immunization were compared with the frequency of splenic human Tregs. As illustrated in Fig. 5b, the titers of human IgM (left panel) and IgG (middle panel) to Pfspz negatively correlated with the frequency of splenic Tregs (− 0.56 and − 0.82, respectively, Spearman correlation coefficient). In contrast, there was no negative correlation between the titers of human IgM to iRBCs and the frequency of splenic Tregs (+ 0.31, right panel). These results suggested that human Tregs play a role in regulating antibody responses to Pfspz antigens but not to iRBC antigens.

### DRAGA mice immunized with CPS-CQ are protected against challenge with Pfspz, but not against challenge with iRBCs

**Fig. 6:**
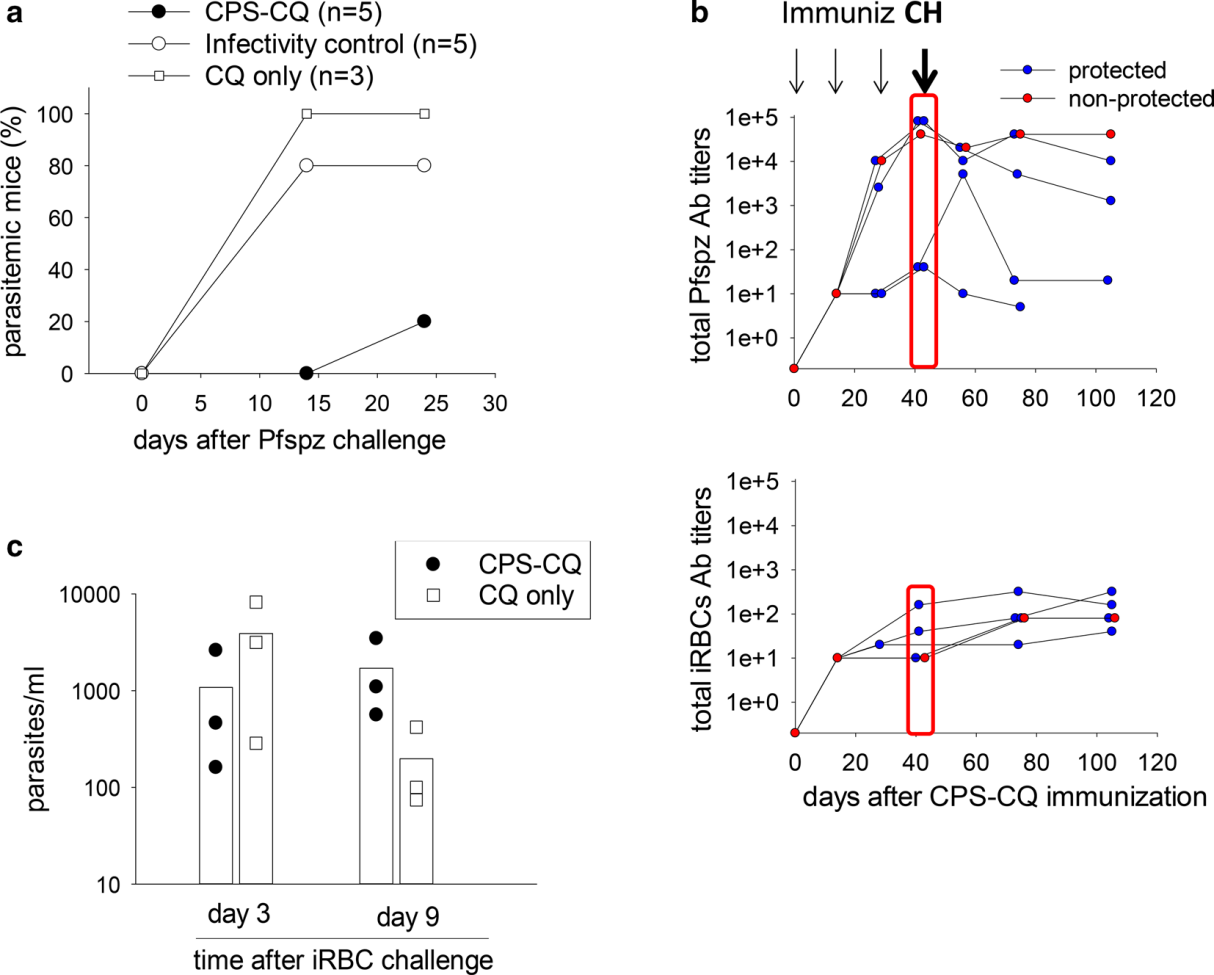
CPS-CQ immunization protects DRAGA mice against challenge with Pfspz, but not against challenge with iRBCs. **a** DRAGA mice (n = 5) were immunized with CPS-CQ three times at 2 weeks interval. Three weeks after the last immunization, mice were challenged intravenously with infectious *P. falciparum* sporozoites (10^5^ per mouse). Control mice were treated with CQ alone (n = 3) or they were untreated (infectivity controls, n = 5) and challenged as above. Mice were followed for blood stage parasitaemia by PCR using primers specific for *P. falciparum* 18S rRNA. Data present the percentage of parasitaemic mice. **b** Kinetics of total human antibody titers to Pfspz (upper panel) and to iRBCs (lower panel) in CPS-CQ immunized mice as in **a**. Antibody titers in protected mice are shown in blue and antibody titers for the unprotected mouse is shown in red. **c** Parasitaemia measured by qPCR using primers specific for *P. falciparum* 18S rRNA in DRAGA mice immunized with CPS-CQ (n = 3) or treated with CQ alone (n = 3) that were challenged intravenously with iRBCs (7 × 10^5^/mouse)

The humanized DRAG mice infused with HLA-matched human HSC reconstitute human hepatocytes (0.02%), Kupffer cells (11%), and human erythrocytes (0.5% haematocrit), and upon challenge with sporozoites they sustain the complete life cycle of *P. falciparum* [10]. Since DRAGA mice immunized with CPS-CQ elicited specific T cell and antibody responses, it was next investigated whether they could be protected against challenge with infectious Pfspz. For this, DRAGA mice (n = 5) were immunized three times with CPS-CQ, and 3 weeks later they were challenged with infectious Pfspz (10^5^/mouse, i.v.). Controls were DRAGA mice treated with CQ only (n = 3) or untreated (infectivity controls, n = 5). Mice were followed for parasitaemia by PCR using primers specific for *Plasmodium* 18S rRNA. As illustrated in Fig. 6a, four out of five CPS-CQ immunized DRAGA mice (80%) did not develop blood stage parasitaemia, while all DRAGA mice treated with CQ (3/3, 100%) and four out of five mice in the infectivity control group (4/5, 80%) became parasitaemic by day 24 after the sporozoite challenge (p = 0.01, Z test). At the time of challenge, all immunized DRAGA mice had developed antibodies to Pfspzs and to iRBCs as measured by IFA, and the antibody titers remained high for up to 9 weeks after the challenge (Fig. 6b). Of note, the only immunized but unprotected DRAGA mouse had similar Pfspz and iRBCs antibodies titers as the immunized and protected mice.

To discern whether CPS-CQ immunization elicited protective pre-erythrocytic or erythrocytic immunity, additional DRAGA mice were immunized with CPS-CQ or treated with CQ alone and 3 weeks after the last immunization they were challenged intravenously with *P. falciparum* iRBCs (7 × 10^5^ per mouse). Blood stage parasitaemia was measured by qPCR using primers specific for *Plasmodium* 18S rRNA at days 3 and 9 post-iRBC challenge. As shown in Fig. 6c, the CPS-CQ immunized DRAGA mice were unprotected against challenge with *P. falciparum* iRBCs since they sustained similar levels of parasitaemia as the control (CQ) mice. In aggregate, these results indicated that protective immunity elicited by *P. falciparum* CPS-CQ immunization in DRAGA mice occurs at the pre-erythrocytic stage of infection.

## Discussion

This study showed that DRAGA mice immunized with *P. falciparum* CPS-CQ elicited human T cell and antibody responses and they were protected against malaria. Protective immunity was pre-erythrocytic as evidenced by the fact that the CPS-CQ immunized DRAGA mice were protected against challenge with Pfspz but they were not protected against challenge with iRBCs. The results in humanized DRAGA mice are in agreement with clinical trials demonstrating that human volunteers immunized with CPS-CQ elicited pre-erythrocytic immunity, since they were protected against challenge with infectious Pfspz [15–19], but were unprotected against challenge with iRBCs [19]. In contrast, studies in rodent malaria models have not reached a consensus on the immunity conferred by CPS-CQ. While BALB/c and C57BL/6 mice immunized with *Plasmodium yoelii* CPS-CQ elicited both pre-erythrocytic and erythrocytic immunity, since they were protected either against challenge with sporozoites or with blood stage parasites [26–28], rats immunized with *P. yoelii* CPS-CQ elicited only pre-erythrocytic immunity [29]. On the other hand, C57BL/6 mice immunized with *Plasmodium berghei* CPS-CQ elicit pre-erythrocytic immunity [30], while C57BL/6 mice immunized with *Plasmodium chabaudi* elicit erythrocytic immunity [31]. The differential ability of CPS-CQ immunization to confer pre-erythrocytic and/or erythrocytic immunity in animals and humans may be explained by intrinsic differences among the many rodent malaria parasites (*P. yoelii*, *P. berghei*, *P. chabaudi*) and *P. falciparum*, as well as differences between the mouse and human immune systems. The results indicating that DRAGA mice immunized with *P. falciparum* CPS-CQ elicited pre-erythrocytic immunity, as found in clinical trials, show the potential of DRAGA mice as a new pre-clinical model to investigate the immunogenicity and protective efficacy of *P. falciparum* malaria vaccine candidates.

This study further revealed that CPS-CQ immunization (i) altered the numbers of human T and B cells in spleen and in the liver, (ii) elicited specific humoral and cellular responses to the Pfspz and to the iRBCs, and (iii) a role of Tregs in regulating humoral responses to Pfspz antigens, but not to the iRBCs antigens.

CPS-CQ immunization in DRAGA mice significantly decreased the numbers of human CD4 and CD8 T cells in spleen and liver with a concomitant increase in the numbers of human B cells. This was most likely related to CQ treatment, rather than to the sporozoite injection, since the same was true for DRAGA mice treated with CQ alone though the small sample size (n = 2 mice treated with CQ alone) is a limitation of the study. However, the reduction in the number of human T cells after CPS-CQ immunization or CQ treatment, as found in this study, is in agreement with data from humans living in malaria endemic areas that were treated with CQ to prevent malaria [32], and with data from patients treated with CQ to ameliorate lupus erythematosus [33], which showed a decreased in the number of human T cells in the peripheral blood. CPS-CQ immunization or CQ treatment also increased the numbers of human B cells in DRAGA mice, though there are no reports from human studies indicating that CQ treatment increases the numbers of human B cells in blood. Of note, this study examined the spleen where the ratio of human T cells as compared to human B cells averages some 1:1, while the human studies examined peripheral blood where the T cells outnumber the B cells (70–85% T cells, 5–20% B cells) [34], which could have masked an effect of CQ for increasing the numbers of human B cells. A safety CPS-CQ clinical trial also reported a reduction on the numbers of human leukocytes in peripheral blood of some of the immunized volunteers, though this human study did not specifically examine the numbers of human T and B cells [18]. Besides the anti-malarial effect of CQ, which is mediated by preventing detoxification of host haemoglobin in the parasite vacuole [35], CQ is also known to induce apoptosis in memory T cells (CD45RA^−^ CD45RO^+^) [36] and to inhibit T cell proliferation in vitro [37, 38]. However, the T cells from CPS-CQ immunized DRAGA were as proficient at secreting human cytokines (IL-2, IFNγ, and TNFα) as the T cells from control (untreated) DRAGA mice, indicating that CPS-CQ immunization did not alter the functional ability of human T cells to secrete cytokines.

Secondly, this study indicated that DRAGA mice immunized with CPS-CQ elicited Pfspz-specific CD4 and CD8 T cells in the liver and spleen, whereas iRBC-specific CD4 and CD8 T cells were mainly localized in the liver. Though this study did not use liver stage parasites for in vitro T cell stimulation due to the difficulty of isolating liver stage parasites [39], it is known that iRBCs express many proteins that are also shared by late liver stage parasites [40]. As such, the iRBCs-reactive T cells found localized in the liver of immunized DRAGA mice might be indeed specific for late liver stage antigens, which would account for the presence of iRBC-specific T cells in the liver. In support of this, it was found that most immunized DRAGA mice had liver-resident human CD8 T cells specific for AMA1, which is a protein expressed by sporozoites, by the late liver stage parasites (schizonts), and by the blood stage parasites [40]. As a note, the DRAGA mice used for this study were well reconstituted with human T cells (Additional file 1: Table S1) and they were proficient at secreting cytokines (Fig. 1c). Though the variability on the malaria-specific human T cell responses developed by each DRAGA mouse immunized with CPS-CQ cannot be explained, humans immunized with CPS-CQ also showed high variability on malaria-specific T cell responses [15–19].

DRAGA mice immunized with CPS-CQ also elicited antibodies against the Pfpsz and to the iRBCs, though the Pfspz antibody titers were significantly higher than those to the iRBCs. This is agreement with data from CPS-CQ clinical trials indicating that while the immunized volunteers elicited antibodies against pre-erythrocytic and erythrocytic antigens, the magnitude of the antibody response was skewed toward the pre-erythrocytic antigens [15, 41]. Furthermore the human B cells of CPS-CQ immunized DRAGA mice secreted IgM and IgG antibodies to the Pfspz but only IgM antibodies to the iRBCs. These results suggest that antibodies to the Pfspz could be T-cell dependent and that antibodies to the iRBCs could be T-cell independent, since helper CD4 T cells are required to support B cell immunoglobulin class switching from IgM to IgG [42], and T-cell independent antibodies are predominantly of IgM isotype [43]. The hypothesis was further supported by the negative correlation between the Pfspz antibody titers and the frequency of splenic human CD4^+^FOXP3^+^ Tregs, whereas there was no negative correlation between the antibodies to iRBCs and the frequency of splenic Tregs. The Tregs are known to regulate antibody responses either (i) by inhibiting helper CD4 T cell function through secretion of suppressive cytokines or direct cell–cell contact interactions [reviewed in 44] or (ii) by direct inhibition on the B cells [25, 45, 46].

This study also suggested that antibodies to the Pfspz or iRBCs (as measured by IFA) may not be sufficient for protection, since the only immunized and unprotected DRAGA mouse had similar Pfspz and iRBCs antibody titers as the protected mice. Studies in rodents that were immunized with *P. yoelii* CPS-CQ indicated that protection is not mediated by antibodies, since transfer of sera from immunized mice into naïve mice did not protect against malaria [26]. In a CPS-CQ human trial in which 2 out 5 immunized volunteers were unprotected, antibodies to CSP and LSA-1 did not significantly differ between protected and unprotected subjects [18]. Furthermore, monoclonal antibodies against the C-terminus of *P. falciparum* CS protein that were generated using B cells of CPS-CQ immunized and protected subjects, showed to be ineffective against malaria infection when tested in mice challenged rodent malaria parasites that express *P. falciparum* CS protein [47].

In aggregate, this study demonstrated that the human immune system of DRAGA mice is robust enough to elicit protective immunity against *P. falciparum*. DRAGA mice thus represent a new humanized mouse model with potential for testing the immunogenicity and protective efficacy of *P. falciparum* malaria vaccine candidates and anti-malarial drugs.

## Conclusions

This study provides first evidence for a *P. falciparum* humanized mouse model (DRAGA) able to elicit protective human immune responses upon malaria vaccination. DRAGA mice immunized with live *P. falciparum* sporozoites and chloroquine elicited systemic (spleen and liver) T cell responses to antigens expressed by the Pfspz and liver T cell responses to antigens expressed by the iRBCs, mounted stronger antibody responses to the Pfspz as compared to the iRBCs, and they were protected against challenge with infectious Pfspz, but unprotected against challenge with iRBCs. The DRAGA mouse model represents a new pre-clinical model that can aid investigating the immunogenicity/protective efficacy of human malaria vaccine candidates and the efficacy of anti-malarial drugs.

## Additional files

**Additional file 1: Table S1.** Human reconstitution in blood of DRAGA mice/percentage of human B and T cells and albumin levels in blood of DRAGA mice; (*), Human hematocrit levels ranked between 0.33 and 0.62%.

**Additional file 2: Figure S1.** Enumeration of human immune cells in DRAGA mice/Mononuclear FSC/SSC FACS gating strategy used to enumerate human cells in DRAGA mice.
