# Supplementary material for: Humanized DRAGA mice immunized with Plasmodium falciparum sporozoites and chloroquine elicit protective pre-erythrocytic immunity
Source: Malar J. 2018 Mar 14;17:114. doi: 10.1186/s12936-018-2264-y (PMC5853061; doi:10.1186/s12936-018-2264-y)
Supplement: Supplementary file 1 — Additional file 1: Table S1. Human reconstitution in blood of DRAGA mice/percentage of human B and T cells and albumin levels in blood of DRAGA mice; (*), Human hematocrit levels ranked between 0.33 and 0.62%. [file 12936_2018_2264_MOESM1_ESM.docx]

*Table S1. Human reconstitution in blood of DRAGA mice*

| **Mouse #*** | **Treatment** | **% human B cells (hCD19^+^)** | **% human T cells (hCD3^+^)** | **Serum human albumin (ng/ml)** |  |
| --- | --- | --- | --- | --- | --- |
| 1 | nil | 16.0 | 22.1 | 117 | Figs 1-5 |
| 2 | nil | 14.2 | 6.7 | 184 |  |
| 3 | nil | 39.4 | 11.2 | 94 |  |
| 4 | nil | 19.7 | 10.1 | 140 |  |
| 5 | nil | 18.6 | 13.7 | 155 |  |
| 6 | nil | 53.8 | 12.9 | 201 |  |
| 7 | nil | 13.7 | 24.4 | 153 |  |
| 8 | nil | 17.7 | 9.7 | 235 |  |
| 1 | CPS-CQ | 19.7 | 66.8 | 51 |  |
| 2 | CPS-CQ | 52.5 | 28.4 | 75 |  |
| 3 | CPS-CQ | 45.2 | 10.1 | 100 |  |
| 4 | CPS-CQ | 42.7 | 11.5 | 104 |  |
| 5 | CPS-CQ | 22 | 14.3 | 68 |  |
| 6 | CPS-CQ | 39.5 | 18.9 | 80 |  |
| 7 | CPS-CQ | 40.6 | 17.5 | 79 |  |
| 1 | CQ only | 39.5 | 18.9 | 225 |  |
| 2 | CQ only | 40.6 | 17.5 | 79 |  |
| 1 | nil | 32.2 | 19 | 40 | Fig 6A |
| 2 | nil | 9.2 | 64.6 | 39 |  |
| 3 | nil | 11.9 | 25.1 | 36 |  |
| 4 | nil | 11.4 | 50.3 | 51 |  |
| 5 | nil | 6.6 | 8.7 | 39 |  |
| 1 | CPS-CQ | 43.9 | 16.1 | 94 |  |
| 2 | CPS-CQ | 40.1 | 8.9 | 66 |  |
| 3 | CPS-CQ | 52 | 5.1 | 59 |  |
| 4 | CPS-CQ | 70.4 | 5.4 | 59 |  |
| 5 | CPS-CQ | 14.7 | 5.9 | 128 |  |
| 1 | CQ only | 2.79 | 7.18 | 98 |  |
| 2 | CQ only | 47.21 | 8.59 | 159 |  |
| 3 | CQ only | 47.2 | 8.6 | 99 |  |
| 1 | CPS-CQ | 39.4 | 3.5 | 162 | Fig. 6C |
| 2 | CPS-CQ | 47.1 | 15.3 | 245 |  |
| 3 | CPS-CQ | 4.1 | 3.8 | 405 |  |
| 1 | CQ only | 26 | 6.6 | 205 |  |
| 2 | CQ only | 11 | 5.3 | 167 |  |
| 3 | CQ only | 23.9 | 3.8 | 429 |  |

Data present the percentage of human B and T cells and albumin levels in blood of DRAGA mice; (*), Human hematocrit levels ranked between 0.33-0.62%
