# Supplementary figures and images for: Humanized DRAGA mice immunized with Plasmodium falciparum sporozoites and chloroquine elicit protective pre-erythrocytic immunity
Source: Malar J. 2018 Mar 14;17:114. doi: 10.1186/s12936-018-2264-y (PMC5853061; doi:10.1186/s12936-018-2264-y)

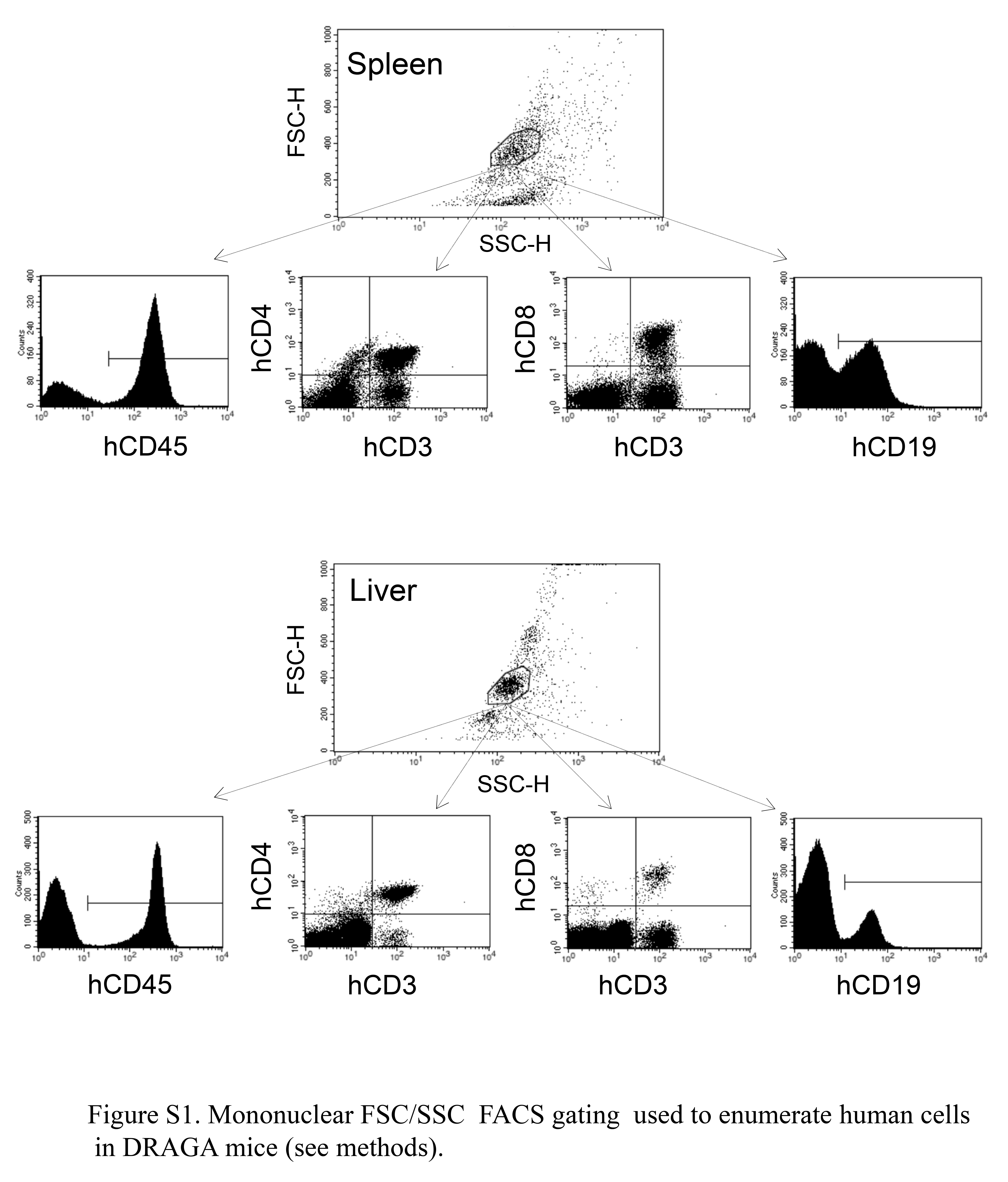

Supplement: Supplementary file 2 — Additional file 2: Figure S1. Enumeration of human immune cells in DRAGA mice/Mononuclear FSC/SSC FACS gating strategy used to enumerate human cells in DRAGA mice. [file 12936_2018_2264_MOESM2_ESM.tif]
